# Data protection and privacy: an introduction

**Published:** 2022-06-07

**Authors:** Elmien Wolvaardt

**Affiliations:** 1 Editor: *Community Eye Health Journal*, International Centre for Eye Health, London School of Hygiene & Tropical Medicine, London, UK.

**Patients’ right to privacy is an important consideration in the design of telemedicine and mobile health initiatives.**

Protecting patients’ personal and medical information is an important part of caring for them. Patients have the right to privacy and the dignity this provides, and will feel more comfortable sharing sensitive, but important, health information if they are confident that their privacy will be protected.

## Principles of data protection

The World Health Organization Regional Office for Europe has developed a useful set of principles for data protection and privacy in health systems^1^ which applies to all forms of telemedicine and mobile health (mHealth). These principles can form a useful starting point for developing your own organisation's policy, while giving due consideration to the laws governing data security in your country and/or region.

According to these principles, patients’ personal data should be:

- processed fairly, in a transparent manner, and with the patient's informed consent (i.e., the patient understands why and how their data will be used and what their rights are)
- obtained and processed only for the purpose of providing health care, as understood by the patient, and not further processed for any other reason
- kept accurate and up to date
- adequate, relevant, and limited to only what is necessary for the purposes of providing health care to the patient
- kept for only as long as is necessary to provide health care to the patient
- kept confidential and not shared with anyone who is not authorised to access it or who doesn't need to access or process it for the purposes of providing health care
- kept safe and secure, protected from accidental loss or alteration.

**From the field**

**Seeing data security from my patients’ perspective**

**Victor Hu**

Consultant Ophthalmologist, Mid Cheshire NHS Hospitals, UK.

The technical details of data governance can often be difficult to remember. What I try to keep in mind is that the important and necessary information we collect about our patients should be kept confidential so that it is only accessed by those who need to and have a good reason to – those who see the patient or are involved with their management. Anyone who isn't involved, or who doesn't have a good reason, shouldn't see any of that patient's information or data.

It is important that we keep patient records and information secure. We don't take patients’ records home as they could easily be lost or stolen. If someone requests information about a patient, we must make sure that the request is genuine, that there is a good reason, that the patient has given consent (if appropriate), and that the information is transferred securely.

If in doubt, I ask myself: “If I were the patient, would I be happy with how my data were being used and stored?” As a health care professional, I should make sure that I can always justify or defend how I am using patients’ information and data and how their information and data are being treated.

**Figure:**
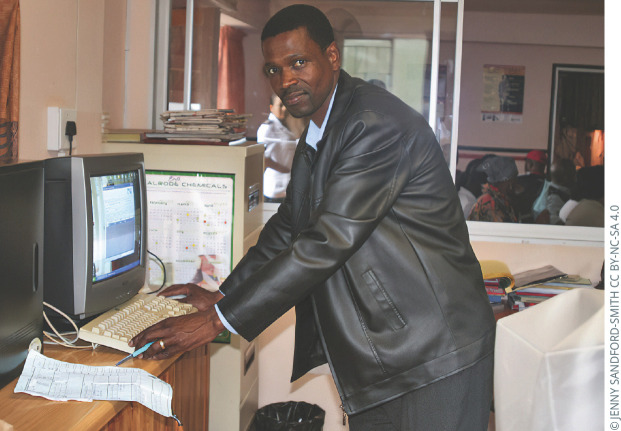
Medical personnel have a responsibility to keep patients’ data confidential and secure. **SWAZILAND**

Patient data should only be transferred to another country if that country can ensure that the patient's data will be adequately protected, as described above.

## Keeping patients’ data safe

Many countries have strict data protection laws. It is important to ensure that health providers know what is expected of them, particularly when storing and transmitting patient information. Putting appropriate measures in place to prevent the loss or theft of personal data is essential for maintaining the trust of patients and the public. This can include:

- encrypting data when it is being stored and/or transmitted
- classifying data, e.g., as strictly confidential, confidential, or public
- managing who has access to which classification of data
- physical security, e.g., keeping files locked and secure and controlling who has access
- setting up a ‘data breach plan’ and communication strategy, with clear allocation of tasks and responsibilities, in the event that there is an accidental or deliberate breach of data security
- assessing and monitoring data security regularly, e.g., by inviting ‘ethical hackers’ to test weaknesses in the system.
